# Methylation of Immune Gene Promoters in Oral and Oropharyngeal Cancer

**DOI:** 10.3390/ijms24097698

**Published:** 2023-04-22

**Authors:** Petra Anić, Jasminka Golubić Talić, Ksenija Božinović, Emil Dediol, Marinka Mravak-Stipetić, Magdalena Grce, Nina Milutin Gašperov

**Affiliations:** 1 Research Department, Srebrnjak Children’s Hospital, 10000 Zagreb, Croatia; 2 Department of Molecular Medicine, Ruđer Bošković Institute, 10000 Zagreb, Croatia; 3 Department of Molecular Biology, Ruđer Bošković Institute, 10000 Zagreb, Croatia; 4 Department of Maxillofacial Surgery, Clinical Hospital Dubrava, 10000 Zagreb, Croatia; 5 Department of Oral Medicine, School of Dental Medicine, University of Zagreb, 10000 Zagreb, Croatia

**Keywords:** oral and oropharyngeal cancer, immune system genes, DNA methylation, HPV

## Abstract

The proportion of oral and oropharyngeal squamous cell carcinoma (OOSCC) that can be attributed to human papillomavirus (HPV) infection is growing nowadays. A potential factor indicating the occurrence of HPV-positive OSCC is a change in the degree of methylation of gene promoters that play a key role in the immune response. In this study, we investigated the difference in the methylation of *EDARADD*, *GBP4*, *HAVCR2*, *HLA DPB1*, *IL12RB1*, *MARCO*, and *SIGLEC12* gene promoters in samples of healthy oral mucosa versus samples of oral and oropharyngeal cancer. The presence of HPV infection in samples was examined earlier. To determine the difference in methylation of those gene promotors, isolated and bisulfite-modified DNA was analysed by the methylation-specific PCR method. The investigated gene promoters were found to be more hypomethylated in the oral and oropharyngeal cancer samples in comparison to normal tissue. The proportion of unmethylated gene promoters was similar in HPV-positive and HPV-negative cancers, although the data should be confirmed on a larger set of samples. To conclude, in samples of healthy oral mucosa, the investigated gene promoters were found to be methylated in a high percentage (73.3% to 100%), while in oral and oropharyngeal cancer samples, they were methylated in a low percentage (11.1% to 37%), regardless of HPV infection.

## 1. Introduction

Head and neck squamous cell carcinoma (HNSCC) is the 6th most frequent malignancy in the world, with over 650,000 new cases diagnosed each year [1]. Unfortunately, despite advances in treatment over the last years, there is still a relapse rate of 50% [2]. The most critical risk factor for HNSCC is excessive tobacco use, with or without alcohol consumption. Moreover, infection with high-risk (HR) types of human papillomavirus (HPV) is being investigated [3]. Because of the bad prognosis and very high mortality of patients, there is a strong need to find new biomarkers of this type of cancer.

Among HNSCC cases, cancer of the oral cavity is the most common, while oropharyngeal squamous cell carcinoma is most often associated with HPV [4], which is the reason for investigating these two types, oral and oropharyngeal squamous cell carcinoma (OOSCC), to a greater extent.

Changes in DNA methylation are possible factors associated with HNSCC, as well as OOSCC, development. DNA methylation, a chemical change that is not mediated by changes in the DNA sequence, is the most widely investigated epigenetic modification [5]. CpG dinucleotides are the target of approximately one-third of the transient changes that occur in human diseases and tumours. To date, the number of CpG sites has been significantly silenced in the mammalian genome by DNA methylation, leading to loss of their function, ultimately to adverse consequences [6].

The host immune system is an essential defence mechanism against tumour development via the coordinated and concerted activation of innate and adaptive immunity. The innate immune response is critical for virus removal [7]. On the other hand, genomic instability and other changes to DNA, including epigenetic ones, cause changes in gene expression and are a common feature of many cancers [8]. Changes in the methylation status of immune system gene promoters lead to a subsequent change in their expression and, thus, point to a possible role in the occurrence and the development of cancer.

Overall, the methylation of different genes, which act in independent pathways, was analysed in a variety of head and neck cancers. Many studies have shown that there are large variations in the choice of the best possible methylation biomarkers and their frequency of DNA methylation in these cancers [9,10]. Several studies have shown that DNA methylation is considered an early event in tumour development, and most investigations have focused on the discovery of biomarkers for early tumour detection [11,12]. Immune dysfunction in OOSCC involves many different phenotypic and functional changes in immune cells that occur with varying frequency in patients with early or late stages of the disease [13]. Hypomethylation in gene promoters normally leads to increased expression and production of proteins encoded by them, while hypermethylation in the same regions causes gene silencing. Hypomethylation in immune system gene promoters allows the production of proteins that have important roles in the defence of the host from foreign organisms, but also potentially in the defence against the development of various tumours, including OOSCC [14].

Current knowledge indicates that DNA methylation, as well as other epigenetic changes, significantly contribute to the appearance of certain types of head and neck cancer (HNC), suppressing or allowing the expression of specific genes that are important in the immune response of the organism itself. Our group conducted several studies on the altered methylation of immune system genes in HPV-related cancers and between a cluster of genes; we assumed that some of them could be validated as possible methylation biomarkers for OOSCC samples. Hence, in this study, we investigated the differences in the promoter methylation status of seven immune system genes, *EDARADD*, *GBP4*, *HAVCR2*, *HLA DPB1*, *IL12RB1*, *MARCO*, and *SIGLEC12*, that could be promising as diagnostic and prognostic biomarkers, and thus in healthy oral mucosa versus oral and oropharyngeal cancers, in order to find out the most eligible biomarkers for those cancers.

## 2. Results

In this study, we investigated the methylation status of immune response gene promotors in 30 oral and oropharyngeal cancer samples and 30 controls (healthy oral mucosa). The subjects differed in age, tumour type, and HPV status. The mean age of the study population was 47 years: 60 years in the cancer patient group and 34 years in the control group. There were 23 oral and 7 oropharyngeal cancers. The HPV status was previously determined [15,16]. Briefly, there were 3 (13.0%) oral cavity cancers, 2 (28.6%) oropharyngeal cancers, and 1 (3.3%) control sample positive for HPV type 16. Three of the cancer samples had DNA inadequate for methylation analysis (two oral and one oropharyngeal cancer), and they were excluded from further analysis. Thus, we examined whether there was a difference in the methylation profiles of seven immune response gene promoters: *EDARADD*, *GBP4*, *HAVCR2*, *HLA DPB1*, *IL12RB1*, *MARCO*, and *SIGLEC12,* between cancer samples and controls. Each tested gene showed some difference in promoter methylation between oral and oropharyngeal cancer tissues and healthy oral mucosal swabs.

Since primers that bind specifically to the unmethylated form of DNA were used in the MSP reaction, in all reactions for all tested genes, we obtained unmethylated control DNA that was positive, while methylated control DNA was negative.

None of the samples of the control group were unmethylated in the *HLA DPB1* and *IL12RB1* gene promoters, from which we can conclude that the methylation in these promoters was 100% of healthy oral mucosa samples. On the other hand, the majority (8/30; 26.7%) of the control samples were unmethylated in the *HAVCR2* gene promoter. A total of 2 samples (6.7%) from this group had unmethylated *GBP4* and *MARCO* gene promoters, while 4 samples (13.3%) were unmethylated in the *EDARADD* and *SIGLEC12* gene promoters (Table 1).

Bisulfite conversion was successful in 27 (21 oral and 6 oropharyngeal cancers) OOSCC tested samples; for 3 samples, no result in MSP reactions was obtained with different primers for methylated (data not presented) or unmethylated forms of individual genes. Most of the oral and oropharyngeal cancer samples were unmethylated in almost all the investigated gene promoters: *EDARADD*, *GBP4*, *HAVCR2*, *HLA DPB1*, *IL12RB1*, *MARCO*, and *SIGLEC12*. The percentage varied between 63% in the case of the *HLA DPB1* gene and 88.9% for the *EDARADD* gene promoter (Table 1).

The majority of the healthy oral mucosa samples (29/30; 96.7%) were HPV-negative; thus, only 1 control sample was HPV-positive for HPV type 16 and was also methylated in all tested gene promoters (*EDARADD*, *GBP4*, *HAVCR2*, *HLA DPB1*, *IL12RB1*, *MARCO*, and *SIGLEC12*). Among the HPV-negative samples, 11/29 (37.9%) were methylated in all the tested genes promoters. None of the samples of this group were unmethylated in the promoters of the *HLA DPB1* and *IL12RB1* gene promoters, from which we can conclude that the methylation in these promoters was obtained in 100% of the healthy oral mucosa HPV-negative or HPV-positive samples.

The methylation of the immune system gene promoters was studied in 27 samples of fresh OOSCC tissue, of which 21 were oral and 6 were oropharyngeal. Of the 27 analysed samples, 5 were HPV-positive; HPV positivity did not affect the gene promotors’ methylation profile. The proportion of HPV-positive cancers that were unmethylated in all tested gene promoters was 60% (3/5). In HPV-negative cancers, the proportion of unmethylated gene promotors was similar; 13 of 22 HPV-negative cancers were unmethylated in the promoters of all tested genes (59.1%). Spearman’s correlation also confirmed no association; the calculated Spearman’s rank correlation coefficient was 0.00719, with the significance level *p* = 0.9716.

## 3. Discussion

The main objective of this study was to determine the difference in the methylation status of the immune gene promoters *EDARADD*, *GBP4*, *HAVCR2*, *HLA DPB1*, *IL12RB1*, *MARCO*, and *SIGLEC12* in healthy oral mucosa versus oral and oropharyngeal squamous cell carcinoma (OOSCC) tissue. Of the investigated immune system genes, the methylation of the HLA class II genes has been studied the most. It has been shown that change in the methylation of *HLA DPB1* (*Major Histocompatibility Complex, class II, DP Beta 1)* contributes to the development of multiple sclerosis, a disease of the central nervous system [17]. Herein, the *HLA DPB1* gene promoter was found to be significantly hypomethylated in cancer tissue (63%) in comparison to healthy oral mucosa tissue (0%). Several studies suggest that DNA methylation in the HLA class II gene region may mediate genetic susceptibility to immune-mediated diseases, such as rheumatoid arthritis [18], type 1 diabetes, and food allergies [19]. The methylation of the *MARCO* gene (*Macrophage Receptor with Collagenous Structure*) has also been investigated in tolerant macrophages. Macrophage tolerance can be induced by a variety of toxic signals, which activate various receptors and signalling pathways. DNA methylation in combination with histone modification is thought to play a role in regulating *MARCO* gene expression. Accordingly, the inhibition with H3K4me3 in the *MARCO* promoter region was significantly reduced after treatment with a demethylating agent [20]. In this study, we found that *MARCO* gene promoter was significantly hypomethylated in cancer tissue (85.2%) in comparison to healthy oral mucosa (6.7%). Studies have shown that hypermethylation of the *IL12RB* (*Interleukin 12 Receptor Subunit Beta*) genes can lead to the reduction of tuberculosis-specific production of the cytokine interferon gamma (IFNγ) [21]. Otherwise, IFNγ expression has an important role in the innate and acquired immune response due to the activation of macrophages and the formation of MHC complexes [22]. We found that *IL12RB1* gene promoter was significantly hypomethylated in cancer tissue (85.2%) in comparison to healthy oral mucosa (0%). In addition, further research is needed to determine whether methylation is an indicator of age itself or if it is functionally related to aging. However, the gene whose methylation is studied with regard to aging is the *EDARADD* (*Ectodysplasin A Receptor Associated Adapter Protein*) gene, whose previously detected methylation in CpG sites was negatively correlated with age, being hypomethylated in older age [23]. We found that *EDARADD* was significantly hypomethylated in cancer tissues (88.9%) in comparison to normal oral mucosa (13.3%), which is in line with age-dependent findings, since the cancer group age (average 60 years) was much higher than that of the normal oral samples. In another study, the hypomethylation of the *EDARADD* promoter was associated with a shorter survival in non-small cell lung cancer compared to those patients who showed higher levels of promoter methylation, and this serves as a biomarker of this disease [24]. A differently methylated region was found in the region of the 5′UTR of the *GBP1* gene (*Interferon-induced Guanylate-Binding Protein 1*), which was hypomethylated in triple-negative breast cancer samples compared to normal samples [25], which is similar to our findings in oral and oropharyngeal cancers. Furthermore, overexpression of *HAVCR* (*Hepatitis A Virus Cellular Receptor*) due to hypomethylation of the gene promoter may be a diagnostic biomarker for colorectal cancer and a prognostic marker for a longer disease-free interval after colorectal cancer surgery [26]. The HAVCR1 protein is also a useful biomarker for diagnosing renal cell carcinoma and ovarian clear-cell carcinoma [27]. We found that *HAVCR2* gene promoter was hypomethylated in 77.8% of oral and oropharyngeal cancers. The *SIGLEC12* gene (*Sialic acid-binding Immunoglobulin-type Lectin 12*) is related to the innate immune system and class I MHC-mediated antigen processing and presentation. We found here that *SIGLEC12* gene promoter was significantly hypomethylated in cancer tissues (85.2%), in comparison to normal oral mucosa (13.3%). Interestingly, the immune system genes were also statistically significantly hypomethylated in cervical cancer (CC) samples compared to normal tissue, and all of the genes analysed in this study belong to the regulatory genes of the immune system [28].

It is known from the literature that there is an association between the hypomethylation of the immune system gene promoters and the presence of HPV. Previous gene expression studies on CC and HNC tissue samples revealed that a number of immune-related genes are dysregulated in HPV-positive cancers compared to normal tissues and HPV-negative cancers [29,30]. Determination of the HPV direct embodiment was recently performed [29,31]. The two most regulated groups of genes were involved in immune regulation and extracellular matrix organization. Subsequently, many of these immune genes were specifically regulated by the HPV type 16 oncoprotein E7, which was desirable to suppress anti-tumour immune responses [31]. Namely, it has been shown that immune system genes are significantly hypomethylated in CC compared to normal tissue, suggesting that immune system activation is most likely a response to HPV oncogenes E6 and E7, which are markedly expressed in advanced cancer [32]. Furthermore, other studies have shown that regulation of host DNA methylation by HPV type 16 E7 is associated with suppression of the host immune system during HPV-associated tumour progression [29]. Interestingly, a recent clinical trial showed that treatment with DNA methylation inhibitors suppressed the growth of HPV-positive HNCs. Significantly, HPV-positive HNCs were more sensitive to treatment with the DNA-demethylating agent 5-aza-2′-deoxycytidine than HPV-negative HNCs [33]. These findings suggest that HPV-induced DNA methylation dysregulation can be reversed by demethylating agents as targeted therapies for tumours associated with HPV infection. Several studies have shown that the presence of HPV DNA is a favourable prognostic factor with respect to relapse and survival [34,35]. In contrast, other studies have shown that patients with positivity to high-risk HPV had a poorer prognosis [36,37] or did not show a significant association between HR HPV infection and clinical outcomes [38,39]. In 2009, Williams et al. investigated whether HPV-specific immune mechanisms could result in tumour removal. For this purpose, they examined immunocompetent and immunologically incompetent mice with or without HPV. In the immunocompetent group, one-third of the HPV-positive mice cleared their tumours compared to the HPV-negative mice. Moreover, the HPV-positive mice had significantly longer survival than the HPV-negative mice [40].

## 4. Material and Methods

### 4.1. Study Group

Healthy oral mucosa samples were collected with a cytobrush from healthy subjects in the School of Dental Medicine, University of Zagreb, Croatia, during regular examinations. Oral cavity and oropharyngeal cancer tissue was collected at the Clinical Hospital Dubrava, Zagreb. The overall study group comprised 60 samples: 30 samples of healthy oral mucosa and 30 OOSCC samples, of which 23 were oral cavity cancers and 7 oropharyngeal cancers. The median age of the control healthy group was 34, while that of the cancer patients was 60. There were 6 (20%) smokers in the healthy group and 23 (76.7%) smokers in the OOSCC samples group. The samples collection was regulated through laboratory service request forms that were signed and approved by the patient and the clinician. The samples were previously tested for mucosal HPV presence, as described earlier [15,16]. Overall, 3 oral cavity cancers (13.0%), 2 oropharyngeal cancers (28.6%), and 1 control sample (3.3%) were positive for HPV (genotype 16).

### 4.2. DNA Preparation

DNA from healthy oral mucosa and OOSCC samples was isolated on BioRobot EZ1 (Qiagen) according to the manufacturer’s instructions. Extracted DNA was dissolved in 50–100 μL of tri-distillate sterile water and then stored at −20 °C. The quality of the samples was evaluated with a NanoPhotometer (Implen, Westlake Village, CA, USA), and those with the ratio A260/280 between 1.7 and 1.9 were included in the study [41].

### 4.3. Bisulfite Conversion

DNA from the OOSCC samples was modified with sodium bisulfite using an EpiTect Bisulfite Kit (Qiagen, Germantown, MD, USA) and purified according to the manufacturer’s instructions. Briefly, after bisulfite treatment, all unmethylated cytosines were converted to uracil, while methylated cytosines remain unchanged.

### 4.4. Genes and Primers Design

Promoters of 7 genes were selected for investigation: *EDARADD* (Gene ID: 128178), *GBP4* (Gene ID: 115361), *HAVCR2* (Gene ID: 84868), *HLA DPB1* (Gene ID: 3115), *IL12RB1* (Gene ID: 3594), *MARCO* (Gene ID: 8685), and *SIGLEC12* (Gene ID: 89858).

The primers’ sequences for unmethylated gene forms were designed with the MethPrimer program for designing bisulfite-conversion-based Methylation PCR Primers in the The Li Lab, Peking Union Medical College Hospital (PUMCH, Beijing, China), Chinese Academy of Medical Sciences.

The primers’ sequences for the bisulfite-converted gene promoters are presented in Table 2.

### 4.5. Methylation Analysis by MSP

The gene promoter methylation status was obtained by the Methylation Specific Polymerase Chain Reaction (MSP) with primers specific for unmethylated forms of 7 gene promoters—*EDARADD*, *GBP4*, *HAVCR2*, *HLA DPB1*, *IL12RB1*, *MARCO*, and *SIGLEC12*—in healthy oral mucosa versus oral and oropharyngeal cancers. Two commercially available whole human genome DNA previously treated with sodium bisulfite (Qiagen, USA) were used as controls: one completely unmethylated (EpiTect Control DNA, unmethylated; Qiagen, USA) and one completely methylated (EpiTect Control DNA, methylated; Qiagen, USA).

MSP was performed for each primer set separately in a 20 µL volume containing 0.4 mM of each dNTP, 1 µM of each primer, 4 mM MgCl_2_, 5× Green GoTaq Flexi buffer, H_2_O, 0.08 µL GoTaq Hot Start Polymerase (Promega, Madison, WI, USA), and bisulfite-converted DNA in a final concentration of 25 ng/µL. All components of the reaction were added in equal volumes, except for the primers and the water, which were added depending on which gene was involved. Methylated and unmethylated control DNA was used in a final concentration of 25 ng/µL. Aliquots of MSP products were analysed on a 2% agarose gel. Briefly, the MSP reaction itself was carried out according to the following conditions: 5 min denaturation at 95 °C was followed by 40 to 45 cycles of denaturation at 95 °C for 30 s, annealing at 56–64 °C for 30 s, elongation at 72 °C for 50 s, and the final 7 min extension at 72 °C.

The annealing temperature varied with different primers: 62 °C for *HAVCR2* (U, Unmethylated primers), 63 °C for *EDARADD* (U) and *GBP4* (U), 61 °C for *SIGLEC12* (U) and *HLA DPB1* (U). At the lowest temperature of 56 °C were bound primers for the *IL12RB1* gene, while at the highest temperature of 64 °C were bound primers for the *MARCO* gene. The number of repeat cycles varied with different primers: 45 for *EDARADD* (U) and 40 for the *GBP4* (U), *HAVCR2* (U), *HLA DPB1* (U), *IL12RB1* (U), *MARCO* (U), and *SIGLEC12* (U) gene primers.

### 4.6. Statistical Methods

The standard Chi-square (χ^2^) test was used for the comparison of two groups of samples (normal vs. cancer) for each gene promoter.

Spearman’s correlation was used, and the Spearman’s rank correlation coefficient between groups’ data (HPV positivity and gene promoter methylation) was calculated.

## 5. Conclusions

In the samples of healthy oral mucosa investigated in this study, it was shown that promoters of genes that have a function in the immune system, such as the *EDARADD*, *GBP4*, *HAVCR2*, *HLA DPB1*, *IL12RB1*, *MARCO*, and *SIGLEC12* genes, are methylated in a high percentage (73.3% to 100%). In OOSCC samples with a different HPV status, it was shown that promoters of the same genes are methylated in a low percentage (11.1% to 37%). However, no correlation with the HPV positivity and the hypomethylation of immune system genes was found. Recently, we have just begun to understand the role of normal DNA methylation in the development and function of the immune system and the role that altered DNA methylation plays in pathological conditions. The demethylation of immune system gene promoters could explain why certain individuals with HNCs have a better prognosis, while others do not. The poorer prognosis of these cancers must almost certainly be related to the fact that these cancers strongly affect the host immune system, and this fact could be important for the development of new therapeutic or prophylactic anticancer approaches.

## Figures and Tables

**Table 1:** Unmethylated immune system gene promoters in healthy oral mucosa and oral/oropharyngeal cancer samples.

Immune System Gene Promoters	No. of Unmethylated Control Samples * (%)	No. of Unmethylated Cancer Samples ** (%)	*p*-Value
*EDARADD*	4 (13.3)	24 (88.9)	*p* < 0.0001
*GBP4*	2 (6.7)	23 (85.2)	*p* < 0.0001
*HAVCR2*	8 (26.7)	21 (77.8)	*p* = 0.0001
*HLA DPB1*	0 (0.0)	17 (63)	*p* < 0.0001
*IL12RB1*	0 (0.0)	23 (85.2)	*p* < 0.0001
*MARCO*	2 (6.7)	23 (85.2)	*p* < 0.0001
*SIGLEC12*	4 (13.3)	23 (85.2)	*p* < 0.0001

* 30 healthy oral mucosa, ** 21 oral and 6 oropharyngeal cancers.

**Table 2:** Primers and primers’ sequences used in the study.

Primer	Sequence 5′-3′
EDARADD-Uf	TTGTTTTGGAATAGTTAGAGGATGG
EDARADD-Ur	AAATACCTAAAAAAATCTCCACACT
GBP4-Uf	TGTGTTTGTAGTTTTAGTTATATGG
GBP4-Ur	TAAAACAAAATTTCACTCTATCACC
HAVCR2-Uf	GGTAGGAGTTTTTAGGGAAAAATG
HAVCR2-Ur	AAACAACAAAATTTATATCCCCATC
HLA DPB1-Uf	GGAAGGTATAGTTTGATTTTGTTTG
HLA DPB1-Ur	AAACCCTATTTATACAAATCCTCATT
IL12RB1-Uf	TTAGTTTGGGTTTAGAGTATGATGT
IL12RB1-Ur	CAAAATAAACTTCTCAAAATACACA
MARCO-Uf	ATTTAGTGTTTGTTAGGATTGTTGG
MARCO-Ur	TAACATCAAAATTTTACCACTCATC
SIGLEC12-Uf	TTGATAATGTAGAAGTTTGTGATGG
SIGLEC12-Ur	ACCAATAACCATAAACTAAATCAAA

Uf = unmethylated forward primer; Ur = unmethylated reverse primer.

## Data Availability

All data are available in the submitted form or upon request.
